# THE LANGUAGE OF LONELINESS: DEFINITIONS, EXPERIENCES, AND CONCEPTUALIZATION: THE VOICE OF MENTAL HEALTH SERVICE USERS

**DOI:** 10.1093/geroni/igae098.0222

**Published:** 2024-12-31

**Authors:** Roger O’Sullivan

**Affiliations:** Institute of Public Health in Ireland, Belfast, Dublin, Ireland

## Abstract

Loneliness is an important public health issue that describes a complex subjective personal experience of one’s desired and actual or perceived social and/or emotional connections. In recent years we have seen a rise in the study of loneliness especially from a quantitative perspective. However, our qualitative understanding of what it means to be lonely and the everyday language best used to capture the personal experience of loneliness remains limited. This presentation based on life story interviews, with older adult mental health service users (18), objectively defined as lonely, gives voice to their definitions, personal experiences and overall conceptualization of loneliness. It sets out a thematic analysis of responses to the question “What does loneliness mean to you? Responses include subjective and objective aspects, frequency, duration and intensity as well as social; emotional and existential loneliness. Loneliness was not defined by participants in a singular manner and was discussed alongside social isolation, depression, grief, as well as feelings of emptiness, boredom and hopelessness. The insights are important to inform how we frame loneliness in research, policy and practice and highlights that our language needs to be sufficiently diverse to capture the complexity of loneliness not only for work with mental health service users but in society as a whole.

